# Mouse Mammary Tumor Virus (MMTV)-Like *env* Sequence in Brazilian Breast Cancer Samples: Implications in Clinicopathological Parameters in Molecular Subtypes

**DOI:** 10.3390/ijerph17249496

**Published:** 2020-12-18

**Authors:** Nathália de Sousa Pereira, Glauco Akelinghton Freire Vitiello, Bruna Karina Banin-Hirata, Glaura Scantamburlo Alves Fernandes, Maria José Sparça Salles, Marla Karine Amarante, Maria Angelica Ehara Watanabe

**Affiliations:** 1 Laboratory of DNA Polymorphisms and Immunology, Department of Pathological Sciences, Biological Sciences Center, Londrina State University, Londrina, PR 86057-970, Brazil; nathalia.pereira2020@uel.br (N.d.S.P.); gvitiello@uel.br (G.A.F.V.); brunakbh@uel.br (B.K.B.-H.); maewat@uel.br (M.A.E.W.); 2 Department of General Biology, Biological Sciences Center, Londrina State University, Londrina, PR 86057-970, Brazil; glaura@uel.br (G.S.A.F.); maze@uel.br (M.J.S.S.)

**Keywords:** breast neoplasia, MMTV, DNA, clinicopathologic parameters, prognosis, virus, carcinogenesis, cancer, epidemiology

## Abstract

**Background:** Breast cancer (BC) is a complex disease in which susceptibility and clinical course depend on multiple factors. Evidence suggests that a mouse mammary tumor virus (MMTV)-homolog may be present in human BCs; however, little is known about its clinical implications. **Methods:** MMTV-like *env* nucleotide-sequence was searched in tumor and tumor-adjacent tissues from 217 Brazilian BC patients through nested-PCR and confirmed through PCR-sequencing. Blood samples were also tested for patients with MMTV-like *env* gene-positive tumors. Correlations with clinicopathological parameters were evaluated. **Results:** MMTV-like *env* sequence was detected in tumor and tumor-adjacent tissue samples from 41/217 and 30/196 patients, respectively. In blood, MMTV-like was detected in 17/32 patients. In Luminal-B tumors, MMTV-like in tumor tissue was negatively correlated with tumor size and disease stage, whereas in HER2 tumors it anti-correlated with lymph node metastasis (LNM) and disease stage. Considering blood, MMTV-like *env* gene positivity negatively correlated with age in general BC, while in Luminal-A tumors it positively correlated with Ki67 but negatively correlated with age and LNM. The associations with decreased LNM frequency were independent of other prognostic factors. **Conclusion:** MMTV-like *env* positivity is associated with better prognostic parameters in BC subtypes, which might be explainable by its anti-metastatic potential and by putative activation of immune milieu.

## 1. Introduction

Breast cancer (BC) is a serious public health issue, considering the number of women who are diagnosed and deaths that occur annually from this disease worldwide [1], and its clinical course and outcome vary from patient to patient depending on a complex series of factors [2,3,4,5,6].

BC can be stratified though the expression of molecular markers, such as estrogen receptors (ER), progesterone receptors (RP), and human epidermal growth factor receptor 2 (HER2) overexpression or amplification, classifying the disease into four major molecular subtypes: Luminal A (LA; ER/PR + HER2-), Luminal-B HER2-positive (LB; ER/PR + HER2+), HER2-enriched (HER2; ER-PR-HER2+) and triple-negative (TN; ER-PR-HER2-) [7,8]. These subtypes have a direct impact on patients’ prognosis and therapeutic response and are used to guide BC treatment [9,10].

Cancers are recognized as multifactorial diseases emerging in the background of multiple genetic and environmental risk factors [11]. Several viruses are known to participate in the induction and in the progression of some neoplasia through different mechanisms, such as expression of oncogenic proteins, induction of cancer-promoting inflammation and insertional mutagenesis. Examples of consolidated oncogenic viruses include the human papilloma virus (HPV) in cervical cancer, the Hepatitis B virus (HBV) in hepatic cancer, the Epstein–Barr virus (EBV) in lymphoma, and the human T-cell Lymphotropic virus (HTLV) in leukemias [12]. Further, other viruses have emerged as potential risk factors for other cancers, including a proportion of human breast tumors [13,14]. 

The mouse mammary tumor virus (MMTV), from *Betaretroviridae* family, was firstly related as a carcinogenic agent by Bittner [15] and is currently considered the most common etiologic agent for BC and T-cell lymphomas in mice [16,17]. Since the discovery of MMTV, researchers have sought to investigate the participation of viruses in human mammary carcinogenesis. These investigations have led to the identification of antigens and sequences homologous to MMTV in human BC tissues by several groups worldwide, indicating that a similar virus, referred to as MMTV-like, might play a role in human BC [18]. Other groups, however, have failed to identify evidence for MMTV-like sequences in human BCs. These discrepancies may be attributable to technical, methodological, or epidemiological variations among studies [18].

Over the last years, several works have narrowed the link between MMTV-like and human BC [18]. Liu et al. [19] were able to isolate a complete provirus from human BC samples and Lawson et al. [20] found MMTV-like antigens and sequences in benign lesions from women that developed BC years later. Furthermore, MMTV-like sequences were found in other human biological samples, such as milk [21], saliva [22], and peripheral blood lymphoid cells [23,24], suggesting that viral transmission and spreading in humans are similar to that described in mice, occurring via breast-feeding and infection of mucosa-associated lymphocytes before reaching the breast tissue [25].

Despite these evidences, there is only one study investigating the presence of MMTV-like sequences in South America, which was conducted in Argentina [26]. Additionally, little is known about the implications of MMTV-like infection on disease prognosis and clinical presentation of BC, especially in BC molecular subtypes. Therefore, the present study aimed to evaluate the presence of MMTV-like *env* gene in breast tissue (tumor and tumor-adjacent tissue) and peripheral blood of Brazilian BC patients and to analyze its correlation with clinicopathological parameters within BC molecular subtypes.

## 2. Materials and Methods

### 2.1. Human Subjects

Following approval from the institutional Ethics Committee for research involving human beings (CAAE 47709015200005231), women diagnosed with BC were invited to participate in the research during the clinical care in the specialized services at Londrina Cancer Hospital (LCH) (Londrina, Paraná, Brazil). A free-informed consent form was signed by all donors prior to sample collection. All patients were treated by the Brazilian Public Health System (SUS).

All patients underwent mastectomy surgery, during which biological samples, consisting of small fragments of freshly excised tissue, were collected by the surgeon responsible from Londrina Cancer Hospital. After collection, the samples were sent to the Laboratory of DNA Polymorphisms and Immunology for processing. In total, fresh tumor tissues were collected from 217 BC patients. Tumor-adjacent tissues and blood samples were also collected at surgery for some of these patients. 

Clinicopathological features retrieved from patients’ medical records included: age at diagnosis, tumor size, histopathological grade, lymph node metastasis (LNM) status, pathological disease stage and immunostaining for Ki67 (cellular proliferation index), p53, estrogen receptor (ER), progesterone receptor (PR) and human epidermal growth factor receptor 2 (HER2). Pathologic disease stage was determined according to the Union for International Control of Cancer (UICC) classification criteria [27]. Immunohistochemical analyses were performed in clinical routine in the Laboratory of Clinical Pathology of the Londrina Cancer Hospital, following standard protocols [28,29].

### 2.2. DNA Extraction

Tumor and tumor-adjacent tissues were mechanically dissociated under aseptic conditions and genomic DNA was obtained through the salting-out method [30]. For peripheral blood samples, DNA was obtained using the Biopur MiniSpin kit (Biometrix diagnostica^®^, Curitiba, PR, Brazil). All DNA samples were quantified in a NanoDrop-2000c Spectrophotometer (Thermo-Fisher Scientific, Wilmington, Delaware, EUA) at 260 nm wavelength. The 260/280 nm absorbance ratio was applied to assess protein contamination and only samples with a minimum of 1.7 ratio were used. DNA samples were stored at −20 °C and adjusted for a concentration of 200 ng/µL with ultrapure water prior to use.

### 2.3. Nested PCR for MMTV-Like env Gene

**Figure 1:**
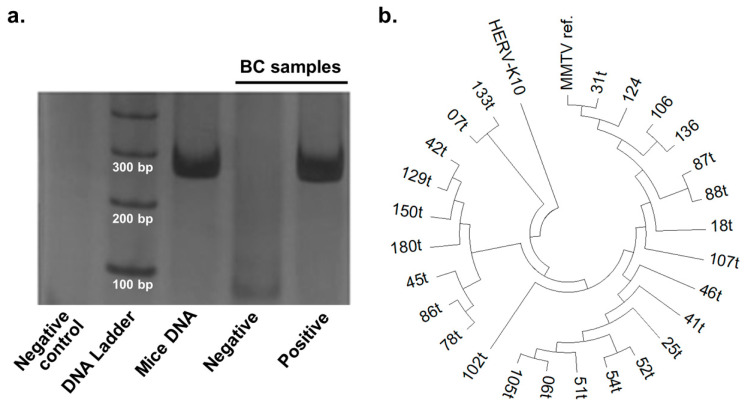
Amplification and sequencing of MMTV-like env sequences. (**a**). Representative picture of polyacrylamide gel after electrophoresis showing a positive and a negative BC sample for MMTV-like *env* amplification along with negative and positive controls (pooled murine mammary gland DNA). (**b**)**.** Phylogenetic tree showing that amplicons from BC samples (represented by numbers) vary in nucleotide sequence and are homologous to MMTV *env* reference sequence (MMTV ref.) but not to HERK-10 reference sequence.

MMTV-like *env* gene sequence was detected by nested polymerase chain reaction (PCR), using the four oligonucleotide primers described by Nartey et al. [21], according to the sequence deposited in the NCBI GenBank (Accession Number KJ831810). PCRs were performed using 16 ng/μL of template DNA in the first PCR round and 2 μL of PCR-product from the first round in the second reaction. In both reactions, reagents concentrations were as follows: 1 × PCR-Buffer (20 mM Tris-HCl pH 8.5, 50 mM KCl), 75 μM dNTP, 0.1 μM of each primer, 1.5 mM MgCl_2_, and 1.25 U Taq DNA polymerase, all from Invitrogen (Carlsbad, California, USA), and ultrapure water to complete a final volume of 25 μL. The reactions took place on a thermal cycler under the following conditions: 95 °C for 5 min followed by 35 cycles of 30 s at 95 °C, 30 s at 58 °C and 30 s and a final extension at 72 °C for 7 min. The amplified products were visualized through electrophoresis in 10% polyacrylamide gels stained with silver nitrate. Positive reactions were represented by the amplification of a 251 bp fragment after the second round of PCR (Figure 1a).

To control for false-positive and false negative results, a negative control without DNA addition and a positive control, consisting of pooled genomic DNA from mammary glands of lactating mice, were also tested in each PCR batch. Furthermore, all DNA samples in the present study were previously used in recent studies by our group [31,32], proving that all samples had amplifiable genomic DNA. In addition, positive samples were independently amplified at least three times and tumor tissues positive for MMTV-like *env* gene were sequenced to confirm the amplification of the target fragment.

### 2.4. Sequencing

Nested-PCR products for MMTV-like *env* were purified using PureLink™ PCR Purification Kit (Invitrogen), following the manufacturer’s instructions. Forward and reverse sequencing reactions were performed in duplicates using the BigDye^®^ Terminator v3.1 kit (Applied Biosystems, Foster City, California, USA), 50 ng of template, and 5 pM of respective oligonucleotide primer in a final volume of 10 μL. PCR conditions were as follows: 10 s at 95 °C, 30 cycles of 20 s at 95 °C, 20 s at 50 °C, and 1 min at 60 °C. The sequencing fragments were analyzed through capillary electrophoresis in a 3500 XL Genetic Analyzer (Applied Biosystems).

The obtained sequences were compared against the NCBI nucleotide databank using the BLAST algorithm. Further, the sequences were aligned and compared with MMTV env (gPr73, accession number 149,186 in NCBI GenBank) and HERV-K10 (accession number AF164613.1 in NCBI GenBank) reference sequences using the MUSCLE algorithm running within MEGA7 software [33] and a phylogenetic tree was constructed using the Tamura–Nei model [34]. 

### 2.5. Statistical Analyses

To analyze the relationship between MMTV-like *env* and clinicopathological features, Kendall’s rank correlation tests were applied, considering Tau-b coefficient for square contingency tables (2 × 2) or Tau-c coefficients for rectangular contingency tables (2 × 3 and 2 × 4). Binary logistic regression analyses were applied to test the association between MMTV-like *env* and the presence of LNM adjusting for tumor size and proliferation index (Ki67). All statistical analyses were two-tailed with a significance level of 0.05 and were performed using SPSS software version 22.0 (IBM^®^, Chicago, IL, USA).

## 3. Results

### 3.1. Prevalence of MMTV-Like env in Breast Cancer Samples

A total of 217 BC samples were included in this work. Patients’ clinicopathological features according to BC subtypes are shown in Table 1.

MMTV-like *env* gene was detected in 18.9% (41/217) of tumor tissues analyzed (Table 2). Of these positive samples, 26 were sequenced and compared to sequences deposited on the NCBI nucleotide bank through the BLAST algorithm, confirming the amplification of MMTV-like *env* gene. Both murine and human sequences returned in the queries with similar scores and identities, confirming that human and murine-derived MMTV-like *env* sequences are genetically indistinguishable.

A representative electrophoretic profile for the nested-PCR to MMTV-like *env* gene is shown in Figure 1a. The obtained sequences were highly similar to the MMTV *env* reference gene, but not to the sequence of HERV-K10 which was previously suggested to be amplified through PCR protocols targeting MMTV sequences [35]. Differences in aligned nucleotide sequences among samples argued against the hypothesis of contamination of PCR reactions or extracted DNA (Figure 1b).

Tumors expressing HER2 showed a higher prevalence of MMTV-like *env* in tumor tissue, with the highest prevalence being observed in tumors from the HER2-enriched subtype (38.5%), followed by those from the Luminal-B subtype (25.0%). TN tumors, otherwise, showed the lower prevalence of MMTV-like *env*, with only one out of 26 samples (3.8%) being positive (Table 2).

Tumor-adjacent tissues were also tested and showed that MMTV-like *env* was present in 15.3% (30/196) samples (Table 2). The frequency of MMTV-like *env* was higher in the tumor than in adjacent tissue in general BC and subtypes, except for TN. Some samples were positive for MMTV-like *env* in adjacent tissue but negative in the tumor tissue. This occurred in 19 of 176 (10.8%) samples in the general group, in 8 of 112 (7.1%) of LA samples, in 3 of 18 (16.7%) LB samples, in 1 of 8 (12.5%) of HER2-enriched samples, and in 4 of 22 (18.2%) of TN samples.

As shown in the literature, MMTV-like sequences were also detected in other biological materials, including peripheral blood. Therefore, we decided to investigate the presence of the MMTV-like *env* in blood samples from patients that were positive in tumor tissue, identifying it in 17/32 (53.1%) of them (Table 2). According to the BC subgroup, MMTV-like *env* prevalence in blood was higher in hormonal-receptor-positive patients (13/24, 54.2%), especially in the LA group, where 57.1% of *env*-positive patients considering breast tissues, were also positive in peripheral blood.

### 3.2. Correlations between MMTV-Like env and Clinicopathological Features

Next, we sought to investigate if MMTV-like *env* presence in tumor tissue was correlated with prognostic clinicopathological features in BC and its subgroups (Table 3). Because of the low prevalence of MMTV-like *env* in TN tumors, correlations could not be analyzed in this subgroup. 

Regarding the presence of *env* in tumor tissue, there was no correlation in general BC group and LA subgroup. However, in LB cancers, it was negatively correlated with tumor size (Tau-c = −0.465; *p* = 0.005) and disease stage (Tau-c = −0.382; *p* = 0.04), and in HER2-enriched BCs it negatively correlated with the presence of LNM (Tau-b = −0.559; *p* = 0.015) and with disease stage (Tau-c = −0.639; *p* = 0.001) (Table 3).

We also sought to investigate the correlation between MMTV-like positivity in blood and clinicopathological parameters. For this purpose, samples that were positive for *env* both in tumor tissue and peripheral blood were compared either with those that were negative for *env* in any tissue or with those that were positive in tumor tissue but not in blood.

In the general BC group *env* positivity in blood was negatively correlated with age considering any group of comparison, and a marked trend towards a negative correlation with LNM (Tau-b = −0.4, *p* = 0.051) was observed when samples positive for MMTV in blood and tumor were compared with samples that were positive only in tumor tissue (Table 4).

In LA subgroup, *env* positivity in blood and tumor tissue was negatively correlated with age at diagnosis and positively correlated with Ki67 when compared to samples that did not amplify the MMTV-like *env* in breast tissue. When considering only LA samples that were positive for the *env* in breast tissue, *env* positivity in peripheral blood was negatively correlated with LNM (Table 4).

### 3.3. MMTV-like is Independently Associated with Lymph Node Metastasis

LNM is a severe latter event in breast carcinogenesis that is associated with poor prognosis. Thus, we investigated whether the associations between MMTV-like *env* and the presence of LNM was independent of other clinicopathological features.

For this purpose, in groups where LNM correlated with MMTV-like *env* in tumor tissue (HER2) or in blood samples (LA), pairwise correlation analyses were performed to identify clinicopathological features correlated with LNM. Then logistic regression models including these factors along with MMTV-like *env* presence were tested.

In HER2+ BCs, no factor other than MMTV-like *env* in tumor tissue was correlated with LNM. Regression models considering only MMTV-like *env* in tumor could not be tested, because all samples positive for MMTV-like *env* in tumor tissue (*n* = 5) were negative for LNM in this group.

In the LA group, LNM was positively correlated with tumor size (Tau-c = 0.312; *p* < 0.001) and Ki67 (Tau-c = 0.185; *p* = 0.029). Therefore, logistic regression models were tested including MMTV-like *env* in blood (compared to tumor tissue *env*^+^ and blood *env*^−^ samples) along with tumor size (Table 5; Model 1), Ki67 (Table 5; Model 2) or both (Table 5; Model 3), as explanatory variables. In all models, MMTV-like *env* was the only factor that remained significant, while tumor size and Ki67 lost significance, indicating that MMTV-like *env* in blood is independently associated with LNM metastasis in this group.

## 4. Discussion

MMTV-like *env* gene was found in varying proportions in human BC tissue worldwide, ranging from 0 to 74% of cases, as analyzed by a recent review by Amarante et al. [18]. In the present study, we found MMTV-like *env* gene in 18.9% of BC tumor tissue in a Brazilian sample, which is similar to the findings from Chinese (16.8%) [36] and Tunisian (13.9%) [37] populations. The only study carried out in South America was conducted in Argentina and has found MMTV-like *env* sequence in 31.0% of BC samples [26]. On the other hand, there are several studies that were not able to evidence the presence of MMTV-like sequences in tumor tissue samples, as shown in studies carried out in Japanese [38] and Iranian [39] populations.

Of note, in the current work, we submitted our PCR amplicons to sequencing, and small variations in nucleotide sequences among samples were observed. Furthermore, the obtained sequences were highly similar both to mouse- and human-derived MMTV *env* sequences in NCBI nucleotide bank, consistent with previous findings [35]. Therefore, the current results are unlikely to be a result of PCR amplification of DNA derived from a single environmental source, as suggested in previous reports [40] and adds new data to previous literature that shows the presence of a virus highly homologous to MMTV in human BC samples with varying prevalence among populations worldwide [18].

Patients positive for MMTV-like *env* in tumor tissue were also tested for the presence of MMTV-like *env* in peripheral blood and MMTV-like *env* was detected in 17/32 (53.1%) of these samples in the general group, and luminal tumors showed the highest proportion of positivity for *env* in blood samples (54.2%). Both results are in accordance with previous studies that identified the presence of MMTV-like sequences in peripheral blood cells of BC patients and showed that it was positively correlated with hormonal receptor expression, which is a hallmark of BC from luminal origin [23].

In previous reports, MMTV-related antigens were found both in T and B cell fractions of peripheral blood from BC patients [24], and active MMTV infection of lymphocytes was shown to be necessary to virus spread to mouse mammary tissue [41], indicating circulating lymphocytes as a source of MMTV-sequences in blood. Additionally, a previous study showed that MMTV-like sequences in blood were positively correlated with distant metastasis [23]. Altogether, these results indicate that hormonal receptors play a role in MMTV presence in the blood, which is conceivably attributable to increased viral production through the activation of estrogen-responsive elements in MMTV-LTR region in mammary cells [42,43,44] and that circulating tumor cells in metastatic tumors may also carry MMTV-like sequences in the blood.

Regarding correlations between MMTV-like *env* in tumor tissue and clinicopathological features, the meta-analysis by Wang et al. [45] reported no association between MMTV-like *env* and expression of ER, PR, HER2, p53 or histological grade. In agreement with these authors, we did not find any correlation between MMTV-like *env* in tumor tissue and clinicopathological parameters from the general BC group. However, in LB and HER2-enriched subtypes, we observed correlations between the presence of MMTV-like *env* gene in tumor tissue and clinicopathological parameters indicative of better prognosis, such as smaller tumor size, lower TNM staging, and lower frequency of LNM. BC molecular subtypes differ in relation to etiology, clinical aspects, and treatment protocols and response [7,8,46]. Our results suggest that MMTV-like may display subtype-specific effects in BC and reinforce the need to consider BC subtypes as separate entities when investigating biomarkers.

Correlations with clinicopathological parameters were also observed for MMTV-like *env* detection in peripheral blood samples. In the general BC and in LA groups, MMTV-like was mostly presented in blood from younger patients. In addition, in LA tumors, MMTV-like in blood correlated with higher proliferation index. We observed that patients who were positive for MMTV-like in tumor tissue and peripheral blood had a significant decrease in the frequency of LNM in comparison to patients that were positive for the *env* gene only in tumor tissue.

LNM is a latter event in carcinogenesis, being indicative of the ability of cancer cells to spread, which is classically related to poor BC prognosis [47,48]. Thus, the relationship between MMTV-like in breast tissue or peripheral blood and LNM was further evaluated by more accurate models, considering the influence of other clinicopathological parameters on LNM.

In HER2-enriched samples, MMTV-like *env* in tumor tissue was the only factor associated with LNM. In LA tumors, otherwise, MMTV-like *env* detection in blood was protective against LNM independently of tumor size and Ki67. It has been shown that MMTV does not encode miRNAs, but alters the expression of host miRNA network by upregulating the expression of oncogenic miRNAs from the miR-17–92 cluster, which has been shown to be a signature of BC, while downregulating the expression of miR-10b-5p, which is associated with metastasis promotion. These results indicate a putative mechanism by which MMTV-like can promote carcinogenesis while inhibiting metastasis [49], which can be consistent with the paradoxical results found in the LA subgroup, in which MMTV-like was positively correlated with proliferation index but negatively correlated with LNM.

Another possible mechanism explaining MMTV-like association with better prognosis in BC might involve the activation of antiviral immune responses in the tumor microenvironment. Indeed, BC shows increased expression of genes related to inflammation and viral infection, such as interferons (IFN) [50]. The immune response provided by type-I IFNs, which are classically activated during viral infections, has been shown to be an important suppressive factor in the metastatic process [51]. Furthermore, several studies have shown that both the expression of endogenous retroviruses or the infection with exogenous viruses indicate enhanced immunogenicity and overall better prognosis in multiple cancer types [52,53,54,55].

The APOBEC3 family of cytidine deaminases are also important components of anti-viral immunity that are induced by IFN signaling. These enzymes work by restricting viral elements through the promotion of mutations in viral nucleic acids. Recent evidence has shown that some of them are endogenous carcinogens by inducing mutations in host DNA in several cancers [56], especially in BC [57]. This mutagenic process, while increasing genomic instability and promoting cellular transformation, shows great potential to generate neoepitopes that can be recognized by the host immune system, being associated with high immunological infiltration in tumor tissue [58]. Strikingly, a germline deletion linking APOBEC3A and APOBEC3B *loci,* which enhances APOBEC3A expression and APOBEC-derived mutagenesis in cancer, has been associated with higher neoepitope loads and immunologic infiltrate [58] and negatively correlated with LNM in BC [59].

Of note, the link between viral infections, APOBEC3 expression, and mutagenesis has been recognized in several virus-associated tumors, such as HPV-positive cervical and oropharyngeal cancers [60] and EBV-positive gastric tumors [61]. Additionally, during MMTV infections in mice, both type I IFNs and APOBEC3 seem to participate in viral restriction [62,63]; and during infection in human breast cells, an APOBEC3G-mediated mutation in *env* is positively selected by increasing of MMTV-like infectivity [64], showing that APOBEC3 cluster is activated during MMTV infection both in mice and human breast cells.

Overall, these evidences indicate mechanisms by which MMTV-like could participate in BC initiation while being associated with favorable prognostic parameters. MMTV-like is a retrovirus and has the potential to upregulate IFN signaling and APOBEC-mediated mutagenesis in BC; and in this way it can increase genomic instability and tumor evolution [57] while promoting cancer cell apoptosis and immunological surveillance through recognition of viral antigens and APOBEC-generated neoepitopes by host immune system [55,58].

## 5. Conclusions

In conclusion, the present work has shown, for the first time, that MMTV-like DNA sequences are present in a proportion BC tissues from Brazilian patients, adding to a growing bunch of epidemiological evidence indicating that MMTV-like DNA sequences are present in human breast tissue of BC patients from different populations worldwide with variable prevalence [18]. Additionally, we were able to detect MMTV-like DNA sequences in peripheral blood samples of BC patients, indicating its potential as a circulating biomarker for BC. In addition, MMTV-like *env* sequence was associated with better prognostic parameters in specific BC subtypes, further strengthening its potential as a valuable marker indicating BC prognosis, beyond being a BC risk factor. Future studies may now focus on the elucidation of possible carcinogenic mechanisms and clinical implications of MMTV-like in human BC, once this knowledge can pave the way for the development of new preventive, prognostic and therapeutic approaches for human BC.

## Figures and Tables

**Table 1:** Patients’ clinicopathological features.

Parameter	General BC (*n* = 217)	Luminal-A (*n* = 139)	Luminal-B (*n* = 24)	HER2 (*n* = 13)	TN (*n* = 26)
**Age (years)**					
Mean (SD)	55.8 (13.1)	56.7 (12.9)	53.1 (10.5)	55.4 (17.8)	55.2 (14.4)
Median (IQR)	54 (19)	56 (19)	49.5 (18)	51.5 (15)	53.5 (22)
<40 [*n* (%)]	23 (10.6)	11 (8.0)	2 (8.3)	3 (23.1)	5 (19.2)
40–49 [*n* (%)]	59 (27.3)	37 (26.8)	10 (41.7)	2 (15.4)	5 (19.2)
50–59 [*n* (%)]	50 (23.1)	32 (23.2)	4 (16.7)	5 (38.5)	6 (23.1)
60–69 [*n* (%)]	50 (23.1)	33 (23.9)	7 (29.2)	1 (7.7)	6 (23.1)
70–79 [*n* (%)]	25 (11.6)	19 (13.8)	1 (4.2)	1 (7.7)	2 (7.7)
>80 [*n* (%)]	9 (4.2)	6 (4.3)	0 (0.0)	1 (7.7)	2 (7.7)
Missed [*n* (%)]	1	1	0	0	0
**Histological Class [*n* (%)]**					
IDC	195 (90.0)	126 (90.6)	22 (91.6)	11 (84.6)	25 (96.2)
ILC	6 (2.8)	4 (2.9)	1 (4.2)	0 (0.0)	1 (3.8)
DCIS	10 (4.6)	5 (3.6)	0 (0.0)	2 (15.4)	0 (0.0)
Other	6 (2.8)	4 (2.9)	1 (4.2)	0 (0.0)	0 (0.0)
**Estrogen/progesterone receptor [*n* (%)]**					
Positive	167 (80.7)	139 (100.0)	24 (100.0)	0 (0.0)	0 (0.0)
Negative	40 (19.3)	0 (0.0)	0 (0.0)	13 (100.0)	26 (100.0)
Missed	10	0	0	0	0
**HER2 [*n* (%)]**					
Positive	37 (18.3)	0 (0.0)	24 (100.0)	13 (100.0)	0 (0.0)
Negative	165 (81.7)	139 (100.0)	0 (0.0)	0 (0.0)	26 (100.0)
Missed	15	0	0	0	0
**Tumor size (cm)**					
Mean (SD)	2.8 (1.9)	2.6 (1.8)	2.8 (2.3)	3.0 (1.5)	3.8 (2.0)
Median (IQR)	2.2 (2)	2.0 (2)	2.2 (1)	2.35 (2)	3.5 (3)
0–1.5 [*n* (%)]	57 (26.6)	40 (29.0)	5 (20.8)	1 (8.3)	4 (15.4)
1.51–3.0 [*n* (%)]	101 (47.2)	69 (50.0)	14 (58.4)	8 (66.7)	6 (23.1)
>3.0 [*n* (%)]	56 (26.2)	29 (21.0)	5 (20.8)	3 (25.0)	16 (61.5)
Missed	3	1	0	1	0
**Histopathological grade [*n* (%)]**					
I	28 (13.7)	24 (18.5)	1 (4.2)	0 (0.0)	0 (0.0)
II	85 (41.7)	63 (48.5)	9 (37.5)	4 (33.3)	6 (23.1)
III	91 (44.6)	43 (33.1)	14 (58.3)	8 (66.7)	20 (76.9)
Missed	13	1	0	1	0
**Ki67 [*n* (%)]**					
Low	48 (25.9)	43 (34.4)	3 (14.3)	0 (0.0)	1 (4.0)
Intermediate	79 (42.7)	60 (48.0)	9 (42.9)	4 (40.0)	5 (20.0)
High	58 (31.4)	22 (17.6)	9 (42.9)	6 (60.0)	19 (76.0)
Missed	32	14	3	3	1
**p53 mutation [*n* (%)]**					
Positive	67 (34.4)	31 (23.5)	11 (55.0)	8 (66.7)	14 (53.8)
Negative	128 (65.6)	101 (76.5)	9 (45.0)	4 (33.3)	12 (46.2)
Missed	22	7	4	1	0
**Lymph node metastasis [*n* (%)]**					
Positive	97 (46.6)	62 (45.3)	12 (54.5)	3 (27.3)	12 (46.2)
Negative	111 (53.4)	75 (54.7)	10 (45.5)	8 (72.7)	14 (53.8)
Missed	9	2	2	2	0
**Tumor stage [*n* (%)]**					
0	10 (5.9)	5 (4.7)	1 (5.3)	2 (15.4)	0 (0.0)
I	36 (21.3)	29 (27.4)	3 (15.8)	2 (15.4)	2 (8.0)
II	69 (40.8)	40 (37.7)	9 (47.4)	6 (46.2)	11 (44.0)
III	44 (26.0)	25 (23.6)	6 (31.6)	2 (15.4)	10 (40.0)
IV	10 (5.9)	7 (6.6)	0 (0.0)	1 (7.7)	2 (8.0)
Unknown	49	33	5	0	1

**Table 2:** Prevalence of Mouse Mammary Tumour Virus (MMTV)-like *env* among BC subgroups.

Breast Cancer Group	MMTV-*env* Frequency [Positive/Tested (%)]		
	Tumor	Adjacent	Blood
General BC	41/217 (18.9)	30/196 (15.3)	17/32 (53.1)
Luminal A	27/139 (19.4)	14/123 (11.4)	12/21 (57.1)
Luminal B	6/24 (25.0)	5/23 (21.7)	1/2 (50.0)
HER2-Enriched	5/13 (38.5)	4/13 (30.8)	1/3 (33.3)
Triple-Negative	1/26 (3.8)	4/25 (16.0)	2/4 (50.0)

**Table 3:** Correlation analyses between MMTV-*env* in tumor tissue and clinicopathological parameters.

BC Group	Parameter	Tau (*p*-Value)
General BC	Age	0.019 (0.764)
	Tumor size	0.012 (0.850)
	Hist. grade	0.048 (0.415)
	Ki67	0.000 (0.994)
	p53	0.084 (0.262)
	LNM	0.008 (0.903)
	Stage	−0.021 (0.753)
Luminal-A	Age	0.017 (0.843)
	Tumor size	0.085 (0.310)
	Hist. grade	0.031 (0.688)
	Ki67	0.028 (0.710)
	p53	0.188 (0.061)
	LNM	0.066 (0.446)
	Stage	0.122 (0.149)
Luminal-B	Age	−0.215 (0.294)
	Tumor size	−0.465 (0.005*)
	Hist. grade	0.097 (0.559)
	Ki67	−0.058 (0.789)
	p53	−0.050 (0.823)
	LNM	−0.158 (0.460)
	Stage	−0.382 (0.042 *)
HER2-enriched	Age	0.355 (0.203)
	Tumor size	−0.222 (0.474)
	Hist. grade	0.111 (0.652)
	Ki67	−0.160 (0.598)
	p53	−0.250 (0.401)
	LNM	−0.559 (0.015 *)
	Stage	−0.639 (0.001 *)

BC: breast cancer; LNM: lymph node metastasis. * *p* < 0.05.

**Table 4:** Correlation between MMTV-like *env* in peripheral blood and clinicopathological parameters.

BC Group	Parameter	Correlation Coefficients [Tau (*p*-Value)]	
		Tu/No^+^Blood^+^ vs. Tu/No^−^	Tu/No^+^Blood^+^ vs. Tu/No^+^Blood^−^
General BC	Age	−0.138 (0.007 *)	−0.480 (0.037 *)
	Tumor size	0.035 (0.526)	0.000 (1.000)
	Hist. grade	−0.021 (0.699)	−0.250 (0.280)
	Ki67	0.062 (0.169)	0.173 (0.468)
	p53	−0.106 (0.220)	0.292 (0.210)
	LNM	−0.061 (0.417)	−0.400 (0.051)
	Stage	0.077 (0.248)	0.132 (0.515)
Luminal-A	Age	−0.187 (0.016 *)	−0.498 (0.095)
	Tumor size	0.084 (0.221)	0.071 (0.808)
	Hist. grade	−0.004 (0.948)	0.000 (1.000)
	Ki67	0.120 (0.042 *)	0.284 (0.262)
	p53	0.192 (0.118)	0.289 (0.259)
	LNM	−0.113 (0.199)	−0.764 (<0.001 *)
	Stage	0.037 (0.653)	−0.015 (0.948)

BC: breast cancer; Tu: Tumor tissue; No: Normal adjacent tissue; LNM: lymph node metastasis. * *p* < 0.05.

**Table 5:** Association between MMTV-*env* in blood and LN metastasis adjusting for other clinicopathological features in luminal A breast cancers.

Factors in the Model	Models Tested		
	Model 1	Model 2	Model 3
**MMTV in blood (*p*-Value)**	0.026 *	0.026 *	0.022 *
Negative	Reference	Reference	Reference
Positive [OR (95%CI)]	0.026 (0.001–0.481)	0.057 (0.005–0.709)	0.028 (0.001–0.600)
**Tumor size (*p*-Value)**	**0.073**	**-**	**0.147**
OR (95%CI)	1.872 (0.944–3.711)	−	2.241 (0.753–6.667)
**Ki67 (*p*-Value)**	**-**	**0.654**	**0.860**
Ki67 low [OR (95%CI)]	−	Reference	Reference
Ki67 Intermediate [OR (95%CI)]	−	2.926 (0.151–56.866)	0.932 (0.034–25.367)
Ki67 High [OR (95%CI)]	−	5.690 (0.139–233.359)	0.184 (0.000–210.232)

OR: odds ratio; CI: confidence interval. * *p* < 0.05.
